# The complete chloroplast genome of *Salsola abrotanoides* (Chenopodiaceae), a desert halophyte shrub in China

**DOI:** 10.1080/23802359.2021.1903353

**Published:** 2021-03-24

**Authors:** Xinyin Li, Qianqian Zhang, Jiecuo Duo, Yuanwu Yang, Xia Ju, Ruijun Duan, Huiyan Xiong

**Affiliations:** a College of Agriculture and Animal Husbandry, Qinghai University, Xining, PR China; b Tsaldam Vocational and Technical College, Delinha, PR China; c College of Eco-Environmental Engineering, Qinghai University, Xining, PR China

**Keywords:** *Salsola abrotanoides*, chloroplast genome, phylogenetic analysis, Chenopodiaceae

## Abstract

*Salsola abrotanoides*, one of the dominant plant species of desert vegetation, adapts well to the arid, saline, and alkaline environment in the Qinghai–Tibetan Plateau. Here, we reported the complete chloroplast sequence and characters of *S. abrotanoides* based on the Illumina NovaSeq Platform. The chloroplast genome is 151,622 bp in length, containing a pair of inverted repeated (IR) regions of 23,701 bp, a large single copy (LSC) region of 84,658 bp, and a small single copy (SSC) region of 19,562 bp. And the chloroplast genome sequence encodes 130 genes totally, including 85 mRNA genes, 37 tRNA genes, and 8 rRNA genes. *S. abrotanoides* is the first species of Genus Salsola and the chloroplast sequence will provide a valuable resource for the phylogenetic studies of Chenopodiaceae.

*Salsola abrotanoides*, which belongs to the family Chenopodiaceae, is widely distributed in Western China (including Qinghai, Gansu, and Xinjiang) and Mongolia. As a halophyte shrub, this species plays an important role on the restoration and reconstruction of desert ecosystem as the main dominant plant in Qinghai–Tibetan Plateau. In recent years, there have been some studies of *Salsola* L. on resistance mechanism such as arid, saline, and alkaline stresses related to photosynthesis special carbon assimilation pathway (C_3_ to C_4_) and water utilization characteristics (Liu et al. 2018; Pyankov et al. 2000; Wen and Zhang 2015; Zhou et al. 2019). However, the chloroplast genome of plant belonged to the genus *Salsola* has not been reported. Here, we focus on the complete plastome sequence of *S. abrotanoides* to provide sufficient resources for genome-wide evolutionary studies and phylogenetic studies of Chenopodiaceae.

In this study, *S. abrotanoides* was collected from Dulan county in the Qaidam Basin, Qinghai Province, China (36.18 N°, 98.50°E) and the fresh, young leaves were frozen in liquid nitrogen immediately and stored at −80 °C. The voucher specimen was deposited at the Herbarium of College of Agriculture and Animal Husbandry, Qinghai University (LK, DLL202001). The whole chloroplast genome of *S. abrotanoides* was characterized by sequencing it using Illumina NovaSeq Platform, assembled with SPAdes version 3.10.1 (Bankevich et al. 2012) and annotated with prodigal version 2.6.3 (Hyatt et al. 2010). At last, the complete chloroplast genome was submitted to GeneBank (GenBank Accession Number: MW123092).

The complete chloroplast genome of *S. abrotanoides* is 151,622bp in length with 36.72% of GC content, containing a pair of inverted repeated (IR) regions of 23,701 bp, a large single copy (LSC) region of 84,658 bp, and a small single copy (SSC) region of 19,562 bp. And the chloroplast genome sequence encodes130 genes totally, including 85 mRNA genes, 37 tRNA genes, and 8 rRNA genes. A total of 244 simple sequence repeats (SSRs) microsatellites were identified and 177 mono-, 3 di-, 60 tri-, and 8 tetra-nucleotide repeats were classified. All the protein-coding genes presented a total of 25,929 codons, with isoleucine (1130 codons, approximately 4.3% of the total codons) as the most abundant amino acid.

The phylogenetic analysis was conducted with an alignment of 21 complete chloroplast genome of Chenopodiaceae family from GenBank, with *Arabidopsis thaliana* as the outgroup, using MAFFT version 7.452 (Katoh et al. 2019) with the FFT-NS-2 strategy. The phylogenetic tree was constructed using neighbor-joining (NJ) tree by MEGA X (Kumar et al. 2018) using 1000 bootstrap replicates (Figure 1). The result showed that *S. abrotanoides* is closely related to genus *Haloxylen* in the phylogenetic tree. The chloroplast sequence will provide a valuable resource for the phylogenetic studies of Chenopodiaceae.

**Figure 1.**
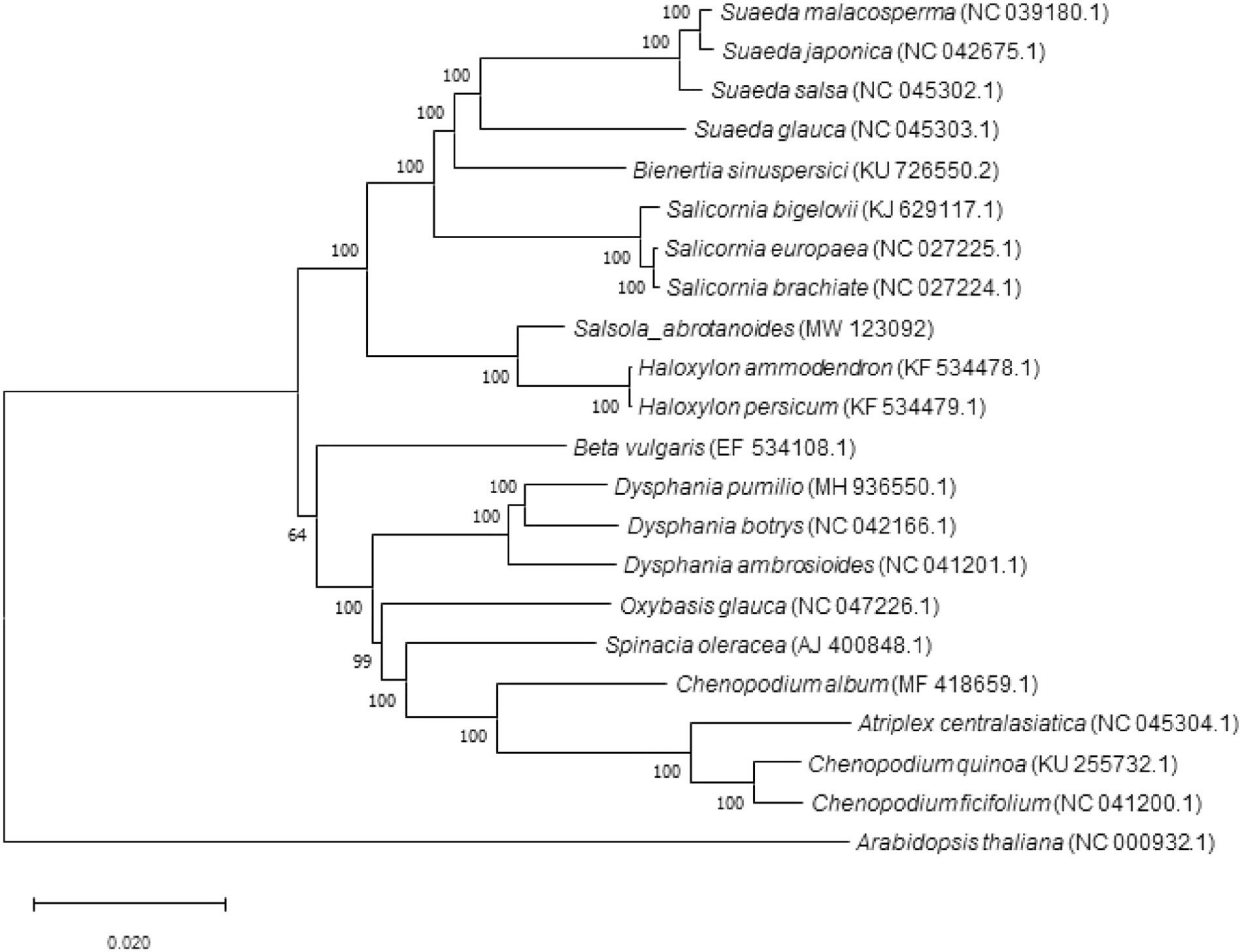
Neighbor-joining tree of 21 Chenopodiaceae species based on the genome sequence. Numbers labeled on the branch are bootstrap values.

## Data Availability

The genome sequence data that support the findings of this study are openly available in GenBank of NCBI at (https://www.ncbi.nlm.nih.gov/) under the accession no. MW123092. The associated BioProject, SRA, and Bio-Sample numbers are PRJNA705397, SRR13805712, and SAMN18087601 respectively.
